# Biotechnological Processes in Microbial Amylase Production

**DOI:** 10.1155/2017/1272193

**Published:** 2017-02-09

**Authors:** Subash C. B. Gopinath, Periasamy Anbu, M. K. Md Arshad, Thangavel Lakshmipriya, Chun Hong Voon, Uda Hashim, Suresh V. Chinni

**Affiliations:** ^1^Institute of Nano Electronic Engineering, Universiti Malaysia Perlis, 01000 Kangar, Perlis, Malaysia; ^2^School of Bioprocess Engineering, Universiti Malaysia Perlis, 02600 Arau, Perlis, Malaysia; ^3^Department of Biological Engineering, College of Engineering, Inha University, Incheon 402-751, Republic of Korea; ^4^Department of Biotechnology, Faculty of Applied Sciences, AIMST University, 08100 Bedong, Malaysia

## Abstract

Amylase is an important and indispensable enzyme that plays a pivotal role in the field of biotechnology. It is produced mainly from microbial sources and is used in many industries. Industrial sectors with top-down and bottom-up approaches are currently focusing on improving microbial amylase production levels by implementing bioengineering technologies. The further support of energy consumption studies, such as those on thermodynamics, pinch technology, and environment-friendly technologies, has hastened the large-scale production of the enzyme. Herein, the importance of microbial (bacteria and fungi) amylase is discussed along with its production methods from the laboratory to industrial scales.

## 1. Introduction

The International Enzyme Commission has categorized six distinct classes of enzymes according to the reactions they catalyze: EC1 Oxidoreductases; EC2 Transferases; EC3 Hydrolases; EC4 Lyases; EC5 Isomerases; and EC6 Ligases [1]. In general, biologically active enzymes can be obtained from plants, animals, and microorganisms. Microbial enzymes have been generally favored for their easier isolation in high amounts, low-cost production in a short time, and stability at various extreme conditions, and their cocompounds are also more controllable and less harmful. Microbially produced enzymes that are secreted into the media are highly reliable for industrial processes and applications. Furthermore, the production and expression of recombinant enzymes are also easier with microbes as the host cell. Applications of these enzymes include chemical production, bioconversion (biocatalyst), and bioremediation. In this aspect, the potential uses of different microbial enzymes have been demonstrated [2–5]. With regard to industrial applications, enzyme purification studies have predominantly focused on proteases, lipases, and amylases [4–12]. Furthermore, several microbes have been isolated from different sources for the production of extracellular hydrolases [5, 13, 14], which are either endohydrolases or exohydrolases. In this overview, we focus on the microbial hydrolase enzyme amylase for its downstream applications in industries and medicines.

## 2. Amylase and Its Substrates

**Figure 1:**
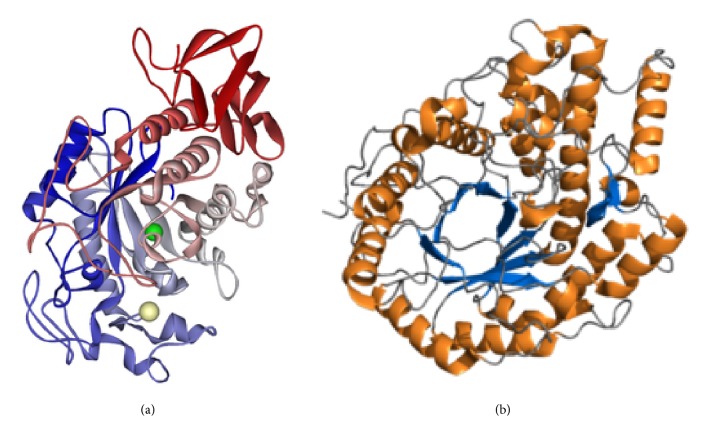
Three-dimensional structures of amylases. (a) *α*-Amylase (RCSB PDB accession code 1SMD; the calcium-binding regions are indicated). (b) *β*-Amylase (RCSB PDB accession code PDB 2xfr).

**Figure 2:**
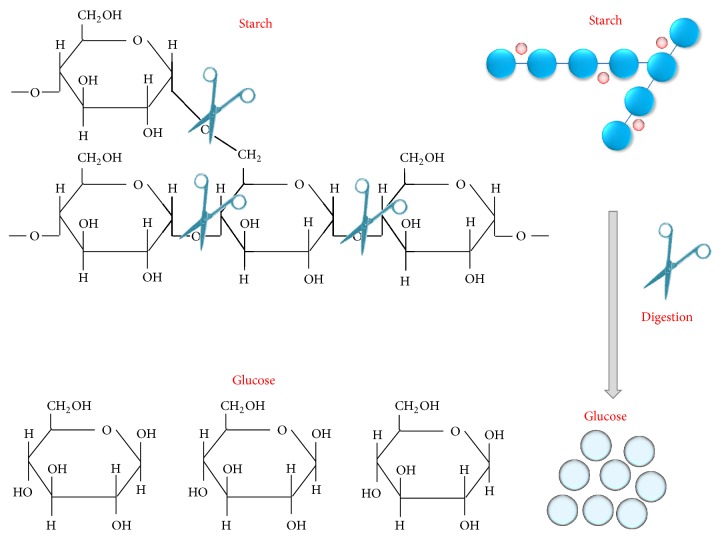
Scheme for the hydrolysis of starch by amylase. Starch is a polysaccharide made up of simple sugars (glucose). Upon the action of amylase, either glucose (a monosaccharide) or maltose (a disaccharide with two glucose molecules) is released.

**Figure 3:**
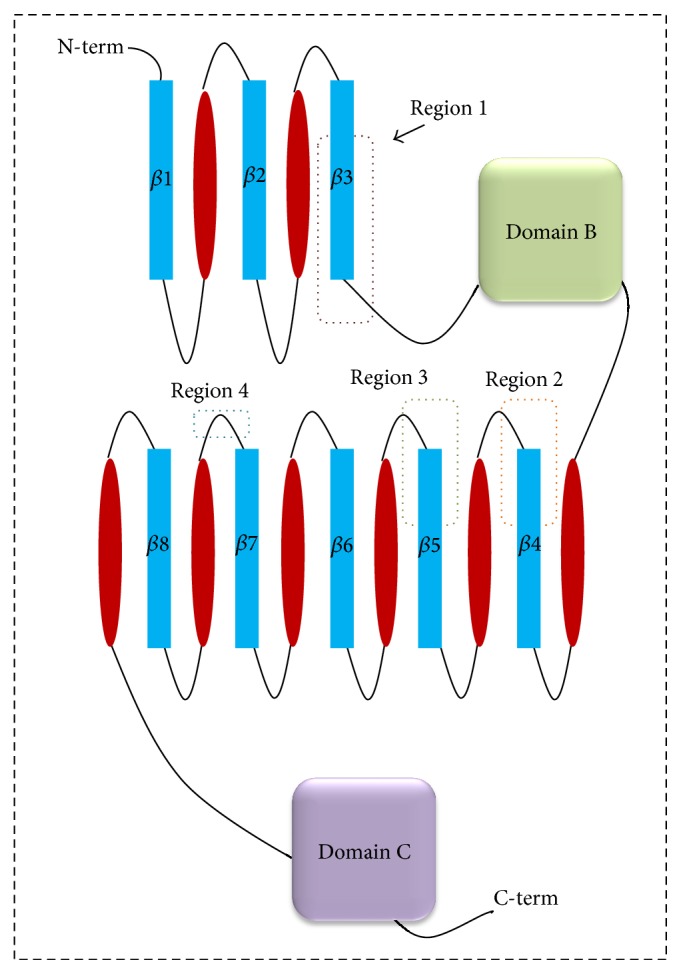
Topology of *α*-amylases. The positions of four conserved sequence patterns are indicated with dashed boxes [18].

Amylases are broadly classified into *α*, *β*, and *γ* subtypes, of which the first two have been the most widely studied (Figures 1(a) and 1(b)). *α*-Amylase is a faster-acting enzyme than *β*-amylase. The amylases act on *α*-1-4 glycosidic bonds and are therefore also called glycoside hydrolases. The first amylase was isolated by Anselme Payen in 1833. Amylases are distributed widely in living systems and have specific substrates [15, 16]. Amylase substrates are widely available from cheap plant sources, rendering the potential applications of the enzyme more plentiful in terms of costs. Amylases can be divided into endoamylases and exoamylases. The endoamylases catalyze hydrolysis in a random manner within the starch molecule. This action causes the formation of linear and branched oligosaccharides of various chain lengths. The exoamylases hydrolyze the substrate from the nonreducing end, resulting in successively shorter end products [16]. All *α*-amylases (EC 3.2.1.1) act on starch (polysaccharide) as the main substrate and yield small units of glucose (monosaccharide) and maltose (disaccharide) (Figure 2). Starch is made up of two glycose polymers, amylose and amylopectin, which comprise glucose molecules that are connected by glyosidic bonds. Both polymers have different structures and properties. A linear polymer of amylose has a maximum of 6000 glucose units linked by *α*-1,4 glycosidic bonds, whereas amylopectin is composed of *α*-1,4-linked chains of 10–60 glucose units with *α*-1,6-linked side chains of 15–45 glucose units. Saboury [17] revealed the *α*-amylases to be metalloenzymes that require metal (calcium) ions to maintain their stability, activity, and structural confirmation. Based on sequence alignments of *α*-amylases, Nielsen and Borchert [18] revealed that these enzymes have four conserved arrangements (I–IV), which are found as *β*-strands 3, 4, and 5 in the loop connecting *β*-strand 7 to *α*-helix 7 (Figure 3). Despite the fact that amylases are broadly available from different sources, past focus has been on only microbial amylases, owing to their advantages over plant and animal amylases, as discussed above. Microbial amylases have been isolated from several stains and explored for amylase production by the methods described below.

## 3. Isolation Methods

The isolation of potential and efficient bacterial or fungal strains is important before being screened for their production of enzymes of interest. As stated elsewhere, microbes are ubiquitous and can be obtained from any source. However, the most efficient strains are usually obtained from substrate-rich environments, from which the microbes can be adopted to use a particular substrate [5, 13]. The common method of strain isolation is through serial dilution, whereupon the number of colonies is minimized and thus easy to select [13]. Another method is through substrate selection, where efficient strains are isolated according to their affinity for a particular substrate [14]. Through these methods, several bacteria and fungi have been isolated and studied for amylase production.

## 4. Microbial Amylase

**Figure 4:**
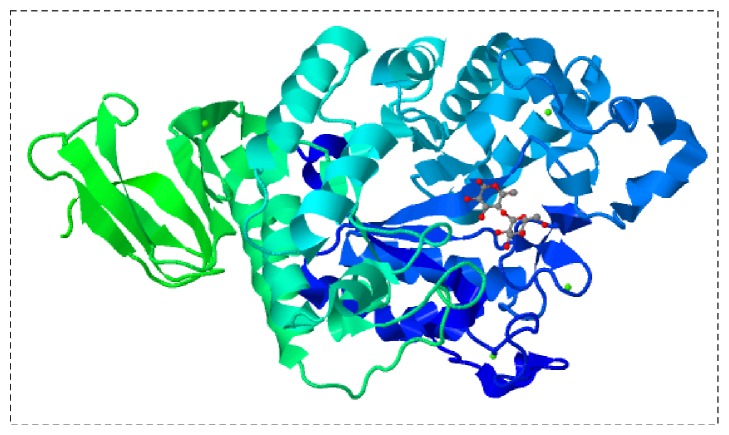
A flowchart for microbial amylase. Three-dimensional structure of the *α*-amylase from* Anoxybacillus *(RCSB PDB accession code 5A2C) [19] is shown.

Microbial amylases obtained from bacteria, fungi, and yeast have been used predominantly in industrial sectors and scientific research. The level of amylase production varies from one microbe to another, even among the same genus, species, and strain. Furthermore, the level of amylase production also differs depending on the microbe's origin, where strains isolated from starch- or amylose-rich environments naturally produce higher amounts of enzyme. Factors such as pH, temperature, and carbon and nitrogen sources also play vital roles in the rate of amylase production, particularly in fermentation processes. Because microorganisms are amenable to genetic engineering, strains can be improved for obtaining higher amylase yields. Microbes can also be fine-tuned to produce efficient amylases that are thermostable and stable at stringent conditions. Such improvements can also reduce contamination by background proteins and minimize the reaction time and lead to less energy expenditure in the amylase reaction [20]. The selection of halophilic strains is also beneficial to the production of amylase under extreme conditions (Figure 4).

### 4.1. Bacterial Amylases

Among the wide range of microbial species that secrete amylase, its production from bacteria is cheaper and faster than from other microorganisms. Furthermore, as mentioned above, genetic engineering studies are easier to perform with bacteria and they are also highly amenable for the production of recombinant enzymes. A wide range of bacterial species has been isolated for amylase secretion. Most are* Bacillus* species (*B. subtilis*,* B. stearothermophilus*,* B. amyloliquefaciens*,* B. licheniformis*,* B. coagulans*,* B. polymyxa*,* B. mesentericus*,* B. vulgaris*,* B. megaterium*,* B. cereus*,* B. halodurans*, and* Bacillus *sp. Ferdowsicous), but amylases from* Rhodothermus marinus*,* Corynebacterium gigantea*,* Chromohalobacter *sp.,* Caldimonas taiwanensis*,* Geobacillus thermoleovorans*,* Lactobacillus fermentum*,* Lactobacillus manihotivorans*, and* Pseudomonas stutzeri *have also been isolated [1, 12, 16, 20, 21]. Halophilic strains that produce amylases include* Haloarcula hispanica*,* Halobacillus *sp.,* Chromohalobacter *sp.,* Bacillus dipsosauri*, and* Halomonas meridiana* [22]. More studies involving the isolation and improvement of novel strains will pave the way to creating important strains. For example, Dash et al. [23] identified a new* B. subtilis *BI19 strain that produces amylase efficiently and, upon optimizing the conditions, enhanced the enzyme production about 3.06 folds. Three-dimensional structural analysis of such amylases helps in improving their efficiency. For example, the crystal structure of *α*-amylase from* Anoxybacillus* has provided insight into this enzyme subclass [19]. Studies on the three-dimensional structure also aid in the alteration or mutation of particular amino acids to improve the efficiency and functions of the enzyme or protein [24–26].

### 4.2. Fungal Amylases

Fungal enzymes have the advantage of being secreted extracellularly. In addition, the ability of fungi to penetrate hard substrates facilitates the hydrolysis process. In addition, fungal species are highly suitable for solid-based fermentation. The first fungal-produced amylase for industrial application was described several decades ago [27]. Efficient amylase-producing species include those of genus* Aspergillus* (*A. oryzae*,* A. niger*,* A. awamori*,* A. fumigatus*,* A. kawachii*, and* A. flavus*), as well as* Penicillium* species (*P. brunneum*,* P. fellutanum*,* P. expansum*,* P. chrysogenum*,* P. roqueforti*,* P. janthinellum*,* P. camemberti*, and* P. olsonii*),* Streptomyces rimosus*,* Thermomyces lanuginosus*,* Pycnoporus sanguineus*,* Cryptococcus flavus*,* Thermomonospora curvata*, and* Mucor *sp. [12, 16, 20, 21].

## 5. Recombinant Amylase

**Figure 5:**
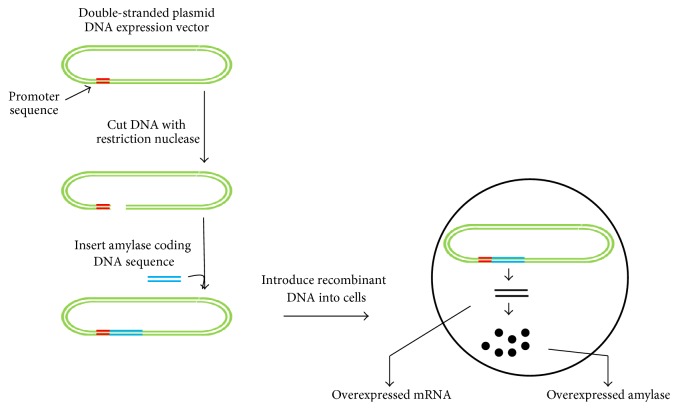
Recombinant DNA technology for amylase production. The steps involve selection of an efficient amylase gene, insertion of the gene into an appropriate vector system, transformation into an efficient bacterial system to produce a higher amount of recombinant mRNA, and overproduction of amylase from the bacterial system.

**Figure 6:**
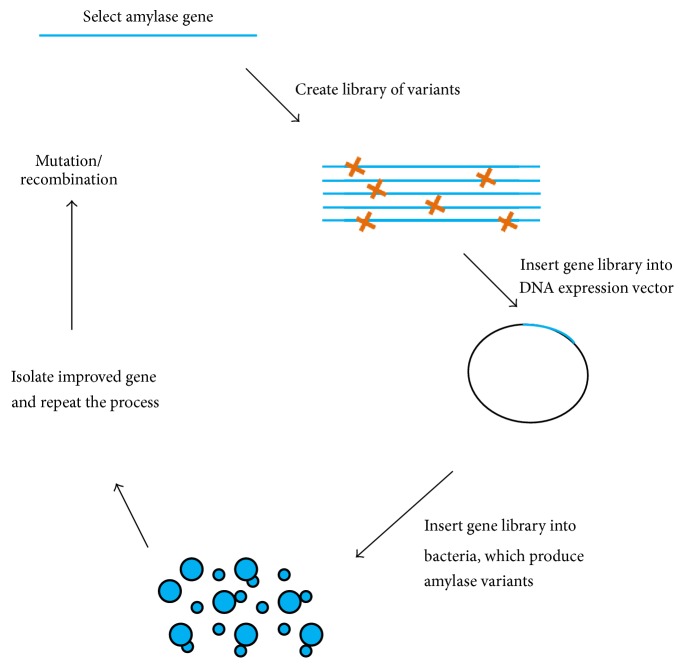
Mutant library screening. Selection of the best variants is a more successful technique for the ultimate application in recombinant amylase production.

Genetic engineering and recombinant DNA technology are the current molecular techniques used to promote efficient enzyme production [18, 28–30]. Recombinant DNA technology for amylase production involves the selection of an efficient amylase gene, gene insertion into an appropriate vector system, transformation in an efficient bacterial system to produce a high amount of recombinant protein (in the presence of an expression-vector promoter-inducing agent), and purification of the protein for downstream applications (Figure 5). In this technology, high-copy numbers of the gene promote higher yields of amylase [30]. On the other hand, screening mutant libraries for selection of the best mutant variants for recombinant amylase production has been more successful (Figure 6). Zhang et al. [31] deleted* amyR* (encoding a transcription factor) from* A. niger* CICC2462, which led to the production of enzyme/protein specifically with lower background protein secretion. Wang et al. [32] generated a new strategy to express the *α*-amylase from* Pyrococcus furiosus *in* B. amyloliquefaciens.* This extracellular thermostable enzyme is produced in low amount in* P. furiosus*, but its expression in* B. amyloliquefaciens* was significantly increased and had good stability at higher temperature (optimum 100°C) and lower pH (optimum pH 5). By mimicking the* P. furiosus* system, they obtained a novel amylase with yields ~3000- and 14-fold higher amylase units/milliliter than that produced in* B*.* subtilis *and* Escherichia coli*, respectively.

## 6. Screening Microbial Amylase Production

**Figure 7:**
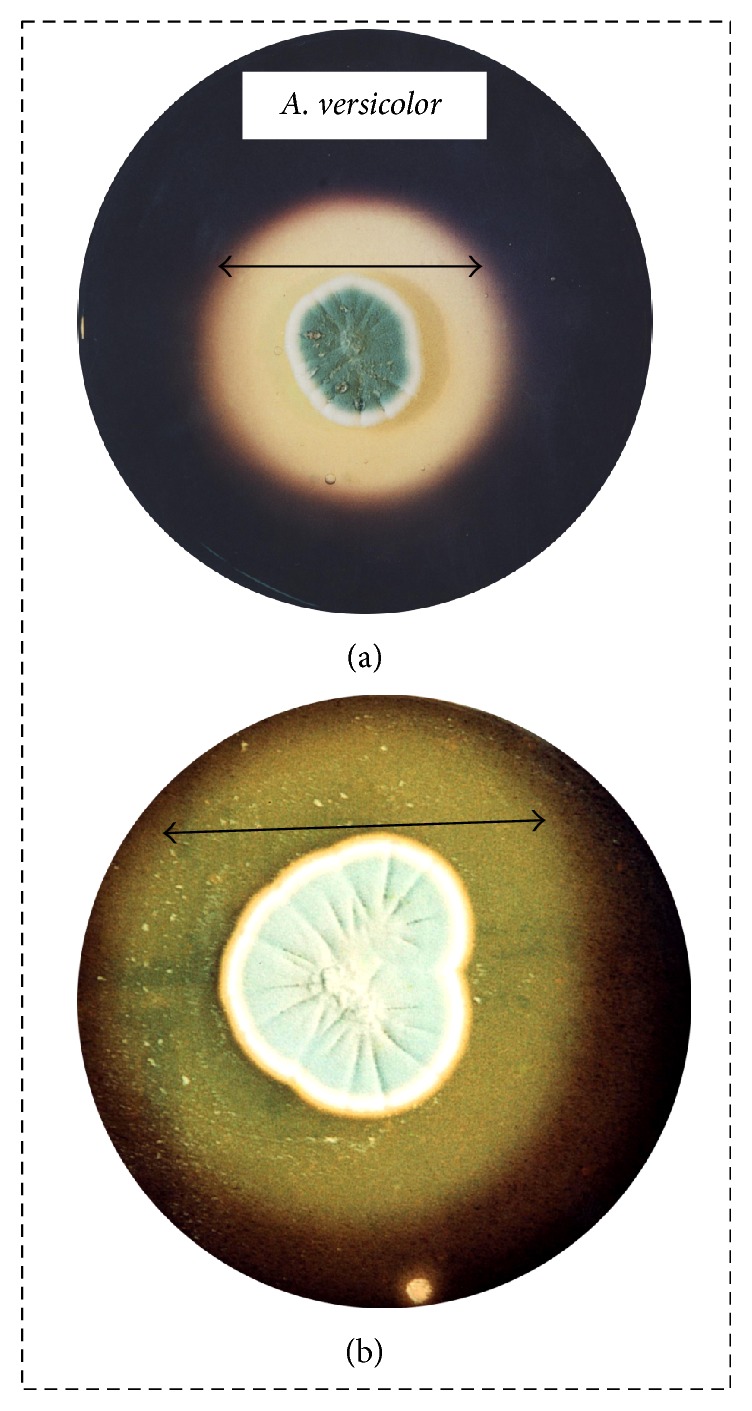
Amylase production on agar plate. In this solid-based method, the starch-containing agar plate is pinpoint-inoculated with the microorganism at the center of the Petri plate. After an appropriate incubation period, flooding the plate with iodine solution reveals a dark bluish color on the substrate region. The clear region around the inoculum indicates the zone of hydrolysis. (a) Amylolytic activity by* Aspergillus versicolor*; (b) amylolytic activity by* Penicillium* sp.

Production or secretion of amylase can be screened by different common methods, including solid-based or solution-based techniques. The solid-based method is carried out on nutrient agar plates containing starch as the substrate, whereas solution-based methods include the dinitro salicylic acid (DNS) and Nelson-Somogyi (NS) techniques. In the solid-agar method, the appropriate strain (fungi or bacteria) is pinpoint-inoculated onto the starch-containing agar at the center of the Petri plate. After an appropriate incubation period, the plate is flooded with iodine solution, which reveals a dark bluish color on the substrate region and a clear region (due to hydrolysis) around the inoculum, indicating the utilization of starch by the microbial amylase. Gopinath et al. [7] applied this method to determine the amylase activity of* Aspergillus versicolor*, as well as that of* Penicillium* sp., in their preliminary study (Figure 7).

In the solution-based DNS method, the appropriate substrate and enzyme are mixed in the right proportion and reacted for 5 min at 50°C. After cooling to room temperature, the absorbance of the solution is read at 540 nm. Gusakov et al. [33] applied this method to detect the release of reducing sugars from substrate hydrolysis by* Bacillus* sp. amylase. They found that the amylase activity could reach up to 0.75 U mL^−1^ after 24 h of incubation. Similarly, in the NS method, amylase and starch are mixed and incubated for 5 min at 50°C. Then, a Somogyi copper reagent is added to stop the reaction, followed by boiling for 40 min and a subsequent cool-down period. A Nelson arsenomolybdate reagent is then added and the mixture is incubated at room temperature for 10 min. Then, after diluting with water, the solution is centrifuged at high speed and the supernatant is measured at 610 nm [34]. Apart from these, several other methods are available for amylase screening, but all use the same substrate (starch).

## 7. Enhancing Microbial Amylase Production

**Figure 8:**
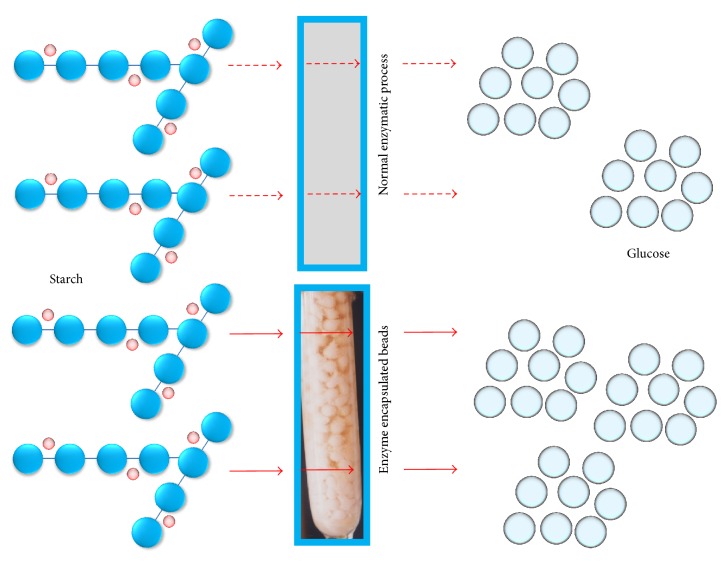
Efficient application of amylase. Differences between the conventional methods of amylase utilization against alginate bead-encapsulated amylase are shown.

The primary objective in amylase production enhancement is to perform basic optimization studies. This can be done either experimentally or by applying design of experiments (DOE) with further confirmation by the suggested experiments from the DOE [35, 36]. Several DOE methods have been proposed and, with the advancement of software, are capable of better predictions [35–38]. Gopinath et al. [8] performed an optimization study by using a Box-Behnken design, involving three variables (incubation time, pH, and starch as the substrate), for higher amylase production by the fungus* A. versicolor*. The laboratory experiments were in good agreement with the values predicted from DOE, with a correlation coefficient of 0.9798 confirming the higher production. Srivastava et al. [37] optimized the conditions for immobilizing amylase covalently, using glutaraldehyde as the crosslinker on graphene sheets. In this study, Box-Behnken-designed response surface methodology was used, with the efficiency of immobilization shown as 84%. This kind of study is important when molecules such as glutaraldehyde are used, owing to two aldehyde groups being available at both ends of the molecule. By optimization study, the chances of immobilizing a higher number of glutaraldehyde molecules can be predicted. In another study, the enzyme-assisted extraction and identification of antioxidative and *α*-amylase inhibitory peptides from Pinto beans were performed, using a factorial design with different variables (extraction time, temperature, and pH) [38]. Another way to enhance the action of amylase is by its encapsulation or entrapment on alginate or other beads (Figure 8). This method facilitates the slow and constant release of enzyme and increases its stability.

## 8. Industrial Applications of Microbial Amylase

Amylase makes up approximately 25% of the world enzyme market [1]. It is used in foods, detergents, pharmaceuticals, and the paper and textile industries [12, 21]. Its applications in the food industry include the production of corn syrups, maltose syrups, glucose syrups, and juices and alcohol fermentation and baking [1]. It has been used as a food additive and for making detergents. Amylases also play an important role in beer and liquor brewing from sugars (based on starch). In this fermentation process, yeast is used to ingest sugars, and alcohol is produced. Fermentation is suitable for microbial amylase production under moisture and proper growth conditions. Two kinds of fermentation processes have been followed: submerged fermentation and solid-state fermentation. The former is the one traditionally used and the latter has been more recently developed. In traditional beer brewing, malted barley is mashed and its starch is hydrolyzed into sugars by amylase at an appropriate temperature. By varying the temperatures and conditions for *α*- or *β*-amylase activities, the unfermentable and fermentable sugars are determined. With these changes, the alcohol content and flavor and mouthfeel of the end product can be varied.

The potential industrial applications of enzymes are determined by the ability to screen new and improved enzymes, their fermentation and purification in large scale, and the formulations of enzymes. As stated above, different methods have been established for enzyme production. In the case of amylase, the crude extract can function well in most of the cases, but for specific industrial applications (e.g., pharmaceuticals), purification of the enzyme is required. This can be accomplished by ion-exchange chromatography, hydrophobic interaction chromatography, gel filtration, immunoprecipitation, polyethylene glycol/Sepharose gel separation, and aqueous two-phase and gradient systems [2], where the size and charge of the amylase determine the method chosen. Automated programming system with the above methods has improved the processes greatly.

With these developments, microbial amylase production has successfully replaced its production by chemical processes, especially in industry [39]. Production of amylase has been improved by using genetically modified strains that reduce the polymerization of maltose during amylolytic action [20]. For further improvement in the industrial process, the above-mentioned DOE and encapsulation methods can be implemented.

## 9. Future Perspectives

Among the different enzymes, amylase possesses the highest potential for use in different industrial and medicinal purposes. The involvement of modern technologies, such as white biotechnology, pinch technology, and green technology, will hasten its industrial production on a large scale. This will be further facilitated by implementation of established fermentation technologies with appropriate microbial species (bacteria or fungi) and complementation of other biotechnological aspects. The technologies of high-throughput screening and processing with efficient microbial species, along with the ultimate coupling of genetic engineering of amylase-producing strains, will all help in enhancing amylase production for industrial and medicinal applications.

## References

[1] Mojsov K. (2012). Microbial *α*-amylases and their industrial applications: a review. *International Journal of Management, IT and Engineering*.

[2] Gopinath S. C. B., Anbu P., Lakshmipriya T., Hilda A. (2013). Strategies to characterize fungal lipases for applications in medicine and dairy industry. *BioMed Research International*.

[3] Gopinath S. C. B., Anbu P., Lakshmipriya T. (2015). Biotechnological aspects and perspective of microbial Keratinase production. *BioMed Research International*.

[4] Anbu P. (2013). Characterization of solvent stable extracellular protease from Bacillus koreensis (BK-P21A). *International Journal of Biological Macromolecules*.

[5] Anbu P., Hur B. K. (2014). Isolation of an organic solvent-tolerant bacterium *Bacillus licheniformis* PAL05 that is able to secrete solvent-stable lipase. *Biotechnology and Applied Biochemistry*.

[6] Gopinath S. C. B., Hilda A., Priya T. L., Annadurai G. (2002). Purification of lipase from Cunninghamella verticillata and optimization of enzyme activity using response surface methodology. *World Journal of Microbiology and Biotechnology*.

[7] Gopinath S. C. B., Hilda A., Lakshmi Priya T., Annadurai G., Anbu P. (2003). Purification of lipase from Geotrichum candidum: conditions optimized for enzyme production using Box-Behnken design. *World Journal of Microbiology and Biotechnology*.

[8] Gopinath S. C. B., Hilda A., Lakshmi Priya T., Annadurai G., Anbu P. (2003). Statistical optimization of amylolytic activity by *Aspergillus versicolor*. *Asian Journal of Microbiology, Biotechnology and Environmental Sciences*.

[9] Kumarevel T. S., Gopinath S. C. B., Hilda A., Gautham N., Ponnusamy M. N. (2005). Purification of lipase from *Cunninghamella verticillata* by stepwise precipitation and optimized conditions for crystallization. *World Journal of Microbiology and Biotechnology*.

[10] Anbu P., Gopinath S. C. B., Hilda A., Lakshmi Priya T., Annadurai G. (2005). Purification of keratinase from poultry farm isolate-*Scopulariopsis brevicaulis* and statistical optimization of enzyme activity. *Enzyme and Microbial Technology*.

[11] Anbu P., Gopinath S. C. B., Hilda A., Mathivanan N., Annadurai G. (2006). Secretion of keratinolytic enzymes and keratinolysis by *Scopulariopsis brevicaulis* and *Trichophyton mentagrophytes*: regression analysis. *Canadian Journal of Microbiology*.

[12] Hussain I., Siddique F., Mahmood M. S., Ahmed S. I. (2013). A review of the microbiological aspect of *α*-amylase production. *International Journal of Agriculture and Biology*.

[13] Gopinath S. C. B., Anbu P., Hilda A. (2005). Extracellular enzymatic activity profiles in fungi isolated from oil-rich environments. *Mycoscience*.

[14] Anbu P., Hilda A., Gopinath S. C. B. (2004). Keratinophilic fungi of poultry farm and feather dumping soil in Tamil Nadu, India. *Mycopathologia*.

[15] Guzmàn-Maldonado H., Paredes-Lòpez O., Biliaderis C. G. (1995). Amylolytic enzymes and products derived from starch: a review. *Critical Reviews in Food Science and Nutrition*.

[16] Gupta R., Gigras P., Mohapatra H., Goswami V. K., Chauhan B. (2003). Microbial *α*-amylases: a biotechnological perspective. *Process Biochemistry*.

[17] Saboury A. A. (2002). Stability, activity and binding properties study of *α*-amylase upon interaction with Ca^2+^ and Co^2+^. *Biologia—Section Cellular and Molecular Biology*.

[18] Nielsen J. E., Borchert T. V. (2000). Protein engineering of bacterial *α*-amylases. *Biochimica et Biophysica Acta*.

[19] Chai K. P., Othman N. F. B., Teh A.-H. (2016). Crystal structure of *Anoxybacillusα*-amylase provides insights into maltose binding of a new glycosyl hydrolase subclass. *Scientific Reports*.

[20] Sundarram A., Krishna Murthy T. P. (2014). *α*-amylase production and applications: a review. *Journal of Applied & Environmental Microbiology*.

[21] de Souza P. M., Magalhaes P. O. E. (2010). Application of microbial-amylase in industry—a review. *Brazilian Journal of Microbiology*.

[22] Kathiresan K., Manivannan S. (2006). *α*-Amylase production by *Penicillium fellutanum* isolated from mangrove rhizosphere soil. *African Journal of Biotechnology*.

[23] Dash B. K., Rahman M. M., Sarker P. K. (2015). Molecular identification of a newly isolated *Bacillus subtilis* BI19 and optimization of production conditions for enhanced production of extracellular amylase. *BioMed Research International*.

[24] Kumarevel T., Nakano N., Ponnuraj K. (2008). Crystal structure of glutamine receptor protein from *Sulfolobus tokodaii* strain 7 in complex with its effector l-glutamine: implications of effector binding in molecular association and DNA binding. *Nucleic Acids Research*.

[25] Kumarevel T., Sakamoto K., Gopinath S. C. B., Shinkai A., Kumar P. K. R., Yokoyama S. (2008). Crystal structure of an archaeal specific DNA-binding protein (Ape10b2) from *Aeropyrum pernix* K1. *Proteins*.

[26] Kumarevel T., Tanaka T., Nishio M. (2008). Crystal structure of the MarR family regulatory protein, ST1710, from *Sulfolobus tokodaii* strain 7. *Journal of Structural Biology*.

[27] Crueger W., Crueger A. (1989). *Industrial Microbiology*.

[28] Corbin J. M., Hashimoto B. I., Karuppanan K. (2016). Semicontinuous bioreactor production of recombinant butyrylcholinesterase in transgenic rice cell suspension cultures. *Frontiers in Plant Science*.

[29] Jung J.-W., Kim N.-S., Jang S.-H., Shin Y.-J., Yang M.-S. (2016). Production and characterization of recombinant human acid *α*-glucosidase in transgenic rice cell suspension culture. *Journal of Biotechnology*.

[30] Son Y. J., Ryu A. J., Li L., Han N. S., Jeong K. J. (2016). Development of a high-copy plasmid for enhanced production of recombinant proteins in *Leuconostoc citreum*. *Microbial Cell Factories*.

[31] Zhang H., Wang S., Zhang X. X. (2016). The amyR-deletion strain of *Aspergillus niger* CICC2462 is a suitable host strain to express secreted protein with a low background. *Microbial Cell Factories*.

[32] Wang P., Wang P., Tian J. (2016). A new strategy to express the extracellular *α*-amylase from *Pyrococcus furiosus* in *Bacillus amyloliquefaciens*. *Scientific Reports*.

[33] Gusakov A. V., Kondratyeva E. G., Sinitsyn A. P. (2011). Comparison of two methods for assaying reducing sugars in the determination of carbohydrase activities. *International Journal of Analytical Chemistry*.

[34] Kobayashi T., Kanai H., Hayashi T., Akiba T., Akaboshi R., Horikoshi K. (1992). Haloalkaliphilic maltotriose-forming *α*-amylase from the *Archaebacterium Natronococcus* sp. strain Ah-36. *Journal of Bacteriology*.

[35] García R., Renedo A., Martínez M., Aracil J. (2002). Enzymatic synthesis of n-octyl (+)-2-methylbutanoate ester from racemic (±)-2-methylbutanoic acid by immobilized lipase: optimization by statistical analysis. *Enzyme and Microbial Technology*.

[36] Chang Y.-N., Huang J.-C., Lee C.-C., Shih I.-L., Tzeng Y.-M. (2002). Use of response surface methodology to optimize culture medium for production of lovastatin by *Monascus ruber*. *Enzyme and Microbial Technology*.

[37] Srivastava G., Singh K., Talat M., Srivastava O. N., Kayastha A. M. (2014). Functionalized graphene sheets as immobilization matrix for fenugreek *β*-amylase: enzyme kinetics and stability studies. *PLoS ONE*.

[38] Ngoh Y.-Y., Gan C.-Y. (2016). Enzyme-assisted extraction and identification of antioxidative and *α*-amylase inhibitory peptides from Pinto beans (*Phaseolus vulgaris* cv. *Pinto*). *Food Chemistry*.

[39] Saranraj P., Stella D. (2013). Fungal amylase—a review. *International Journal of Microbiological Research*.

